# Complete Genome Sequence of *Staphylococcus epidermidis* 1457

**DOI:** 10.1128/genomeA.00450-17

**Published:** 2017-06-01

**Authors:** Madeline R. Galac, Jason Stam, Rosslyn Maybank, Mary Hinkle, Dietrich Mack, Holger Rohde, Amanda L. Roth, Paul D. Fey

**Affiliations:** a Multidrug-Resistant Organism Repository and Surveillance Network (MRSN), Walter Reed Army Institute of Research, Silver Spring, Maryland, USA; b Institut für Medizinische Diagnostik GmbH, Bioscientia Labor Ingelheim, Ingelheim, Germany; c Institut für Medizinische Mikrobiologie, Virologie & Hygiene, Universitätsklinikum Hamburg-Eppendorf, Hamburg, Germany; d Department of Pathology and Microbiology, University of Nebraska Medical Center, Omaha, Nebraska, USA

## Abstract

*Staphylococcus epidermidis* 1457 is a frequently utilized strain that is amenable to genetic manipulation and has been widely used for biofilm-related research. We report here the whole-genome sequence of this strain, which encodes 2,277 protein-coding genes and 81 RNAs within its 2.4-Mb genome and plasmid.

## GENOME ANNOUNCEMENT

*Staphylococcus epidermidis* is a commensal bacterium that colonizes human skin and mucous membranes (1, 2). Its commensal function is not well understood but clearly involves inhibition of pathogen colonization (3). However, due to its proximity to the insertion sites of catheters or other foreign bodies, *S. epidermidis* frequently colonizes these devices and forms biofilms that are inherently resistant to the host immune system (4) and antibiotics (5). Most infected foreign devices require removal from the body for effective treatment, resulting in significant morbidity.

*S. epidermidis* strain 1457 (6) is often used as the model organism for molecular studies investigating biofilm formation for multiple reasons. First, strain 1457 is pansusceptible to antibiotics, including erythromycin, the most common antibiotic used as a marker in staphylococcal genetics. Second, in contrast to many *S. epidermidis* strains, including RP62A (7), strain 1457 is amenable to genetic manipulation, including transduction with Φ71 (8), A6C (9), and Φ187 (10). Third, strain 1457 produces a significant amount of polysaccharide intercellular adhesin-dependent biofilm (11, 12) and thus is an excellent model strain to understand *icaADBC* transcriptional regulation. Additionally, accumulation associated protein-dependent biofilm formation has also been studied in this strain using *icaADBC* allelic replacement mutants (13, 14). Finally, multiple allelic replacement mutants already exist for strain 1457 (15–28).

Sequencing was performed as previously described (29). RS II (Pacific Biosciences, USA) single-molecule real-time sequencing (SMRT) produced 81,634 reads with an average length of 15,543 bp. The reads were assembled using HGAP2 in the SMRT Analysis Portal into two polished contigs, one for the chromosome and one for the plasmid, p1457. MiSeq (Illumina, Inc., USA) short-read sequencing produced 1,526,588 reads with an average length of 350 bp and insert size of 500 bp. These reads were mapped to the SMRT sequences using the mapper within Geneious (Biomatters, New Zealand), resulting in an average depth of coverage of 199×. Genes were predicted using the NCBI Prokaryotic Genome Annotation Pipeline version 4.1.

The genome of strain 1457 is 2,454,929 bp long containing 2,260 protein-coding sequences (CDSs) and 81 RNAs with a 32.3% GC content. The plasmid, p1457, comprises 15,142 bp coding for 17 CDSs.

A previous draft genome for this strain, which was sequenced by another group and comprises 2,417,500 bp in 74 contigs, is available in GenBank (accession no. JMID00000000.1). We found that 73 draft contigs aligned to the finished genome, while the remaining one aligned to the plasmid. This alignment demonstrates the challenges of short-read assembly, with the draft contigs ending in genes known for being highly variable or typically present with multiple copies. Additionally, there are genes that appear to be missing between the draft contigs. We identified minimal differences between the two versions, which we were confident were not the result of assembly errors: two intragenic amino acid substitution single nucleotide polymorphisms (SNPs), one intergenic SNP, and one intragenic insertion in the draft relative to the finished genome.

### Accession number(s).

The complete genome sequence of *S. epidermidis* 1457 has been deposited in GenBank under the accession numbers CP020462 to CP020463.
